# Platelet-Rich Plasma Guided Injections: Clinical Application in Peripheral Neuropathies

**DOI:** 10.3389/fsurg.2014.00041

**Published:** 2014-10-13

**Authors:** Michael-Alexander Malahias, Dimitrios Chytas, George C. Babis, Vasileios S. Nikolaou

**Affiliations:** ^1^2nd Orthopaedic Department, Athens University, Athens, Greece

**Keywords:** platelet-rich plasma, clinical studies, peripheral neuropathies, evidence, review

## Abstract

Platelet-rich plasma (PRP) is defined as an autologous concentrated preparation of platelets and their associated growth factors in a small volume of plasma. The presence of these growth factors has stimulated the scientific community to search about possible benefits of the use of PRP in tissue regeneration. Provided that previously *in vitro* and animal research demonstrated that PRP could probably play an important role in the treatment of neural tissue disorders, we aimed to review the current literature, regarding the clinical studies that have been conducted to confirm this hypothesis. More specifically, we have reviewed the literature concerning the clinical application of PRP in peripheral neuropathies and investigated if there is strong evidence to establish the use of PRP in clinical practice as a therapeutic option. In contrast with animal studies, we have been able to identify only few clinical data concerning the use of PRP in peripheral neuropathies. We found five trials matched to our research that yields positive and promising results for the future for the application of PRP for the therapy of disorders of the peripheral nervous system. It is obvious that this interesting field of research gives to the scientists the ability to expand it extensively, in terms of both quality and quantity.

## Introduction

Platelet-rich plasma (PRP) is an innovative and promising approach in tissue regeneration. PRP is defined as an autologous concentrated preparation of platelets and their associated growth factors in a small volume of plasma (1). Platelets are a natural source of a myriad of growth factors in their natural and biologically determined ratios (2).

The application of PRP has been documented in many fields. Musculoskeletal practitioners began using PRP for chronic tendinopathy in the early 1990s. The growth of PRP therapy has relied primarily on anecdotal or case studies. Historically, there have been few controlled trials to prove the efficacy of PRP. Recently, however, there has been an emerging literature on the beneficial effects of PRP for tendon, ligament or muscle injuries, maxillofacial therapies, dentistry, cosmetics, wound healing, cardiothoracic surgery, ear–nose–throat surgery, neurosurgery, urology, and ophthalmology.

Among all these data, a relatively new question upon basic science’s researchers is the effect of PRP on neural tissue. There is an important number of animal studies that argue in favor of the use of PRP in neuropathies. Most recently, Giannessi et al. (3) experimented with an autologously generated PRP suturable membrane in sciatic nerve neurotmesis–neurorrhaphy (rat model). He concluded that PRP may enhance peripheral nerve regeneration. Farrag et al. (4) showed in a rat model that there is an improved functional outcome with the use of PRP – in comparison with no bioactive agents [platelet-poor plasma (PPP)] – on facial nerve regeneration. In another study, Park and Kwon (5) proved that PRP limits the nerve injury caused by 10% dextrose in the rabbit median nerve (carpal tunnel model). On the other side, Piskin (6) suggested that platelet gel does not improve peripheral nerve regeneration, but Kaplan (7), from the same team, 2 years later, observed that platelet gel has a positive effect on sciatic nerve repair (animal studies). In different fields, Elgazzar (8), Sariguney (9), and Shen (10) concluded that PRP can be a novel treatment for peripheral or central nervous system diseases.

### But how could PRP possibly react to nerve tissue?

Platelets include alpha granules, which contain mitogenic and chemotactic growth factors such as IGF-1, bFGF, and TGF-beta (11). Although they are not classical neurotrophic factors, the effects of these growth factors on nerve regeneration have been comprehensively studied.

About nerve entrapment syndromes, Mulvaney (12) introduced a new theory about non-surgical treatment. He showed that peripheral nerve entrapments can be treated with real time ultrasound-guided hydro-neurolysis. Hydrodissection around the entrapped nerve could possibly work to loosen the scar tissue allowing more nerve impulses to pass through. Independent of the fluid (PRP or local anesthetic or corticosteroid or normal saline 0.9%), hydrodissection works in a mechanical and non-biochemical way, but it maybe improves cases of ischemic damage to a nerve due to scar tissue banding.

Giannessi (3) believes that the use of PRP could perform an action, not only as a source of bioactive proteins, but also as a nerve guide to hold the scar reaction and thus induce axonal regeneration.

Provided that the previously mentioned *in vitro* and animal research demonstrated that PRP could probably play an important role in the treatment of neural tissue disorders, we aimed to review the current literature, regarding the clinical studies that have been conducted to confirm this hypothesis. More specifically, we have reviewed the literature concerning the clinical application of PRP in peripheral neuropathies and investigated if there is strong evidence to establish the use of PRP in clinical practice as a therapeutic option.

## Literature Review

We searched on Medline, Cochrane Database, and EMBASE using the key words “PRP,” “clinical study,” “peripheral neuropathy,” and “trial.” Initially, one reviewer conducted the literature search and retrieved the references to be evaluated. A second reviewer independently selected the trials to be included in the review and also screened the reference list from the selected articles in order to identify studies that have been missed at the initial search.

In contrast to animal studies, we have been able to identify only few clinical data concerning the use of PRP in peripheral neuropathies. We found five trials matched to our research.

According to Anjayani et al. (13), PRP injection could promote improvement of peripheral neuropathy in patients with Hansen’s disease. This double-blind, randomized, clinical trial included 60 patients with neuritis leprosy (Hansen’s disease). This kind of nerve damage, caused by *Mycobacterium leprae*, is characterized by granulomatous inflammation of the epineuron of peripheral nerves. The sensory loss appears to be earlier and more frequent among patients. In this research, patients were divided randomly in two comparable groups and treated either with a PRP perineural injection or a PPP injection as control. Perineural injection of PRP proved to be significantly more effective in visual analog scale (VAS) and two-point discrimination test. This result is in favor of the positive effect of PRP in nerve regeneration. However, the conclusions of this high-quality study need to be confirmed with further research in the future, with longer observation times, while, on the other hand, the optimal frequency of PRP injections should be determined (13).

Sanchez et al. (14) described a case report of a healthy young man who suffered peroneal nerve palsy with drop foot after multiple ligament injuries of the knee. In this case, the patient had already undergone a conservative treatment for 11 months without any improvement. Then, with the use of ultrasound guidance, he was treated with intraneural injections of plasma, rich in growth factors. At the follow-up period of 21 months, he was assessed by electromyography (EMG), ultrasound echogenicity, and manual muscle testing. At the end of this period, the patient expressed partial recovery, so that he was satisfied with running without orthosis. EMG study showed complete reinnervation for the peroneallongus and a better reinnervation for anterior tibialis muscle, compared with previous examination. Sensitivity of the peroneal nerve distribution area was also fully recovered. This case report introduces a novel technique of management of nerve palsy, but, according to the authors, more extensive research is required for the clarification of the mechanism of action of PRP. Finally, the authors’ hypothesis that the clinical outcome might be better with earlier application of their method, before muscle degeneration occurs, needs to be confirmed with more studies in the future (14).

In another case study from Kuffler et al. (15), a long ulnar nerve gap was repaired more than 3 years after the initial trauma. This report investigated whether a collagen tube filled with autologous platelet-rich fibrin could induce sensory and motor recovery. Two years after the operation, the patient showed good ring and small finger motor function (could elevate 1 kg of weight). Also, he had topographically correct two-point discrimination and ability to feel vibration. The patient’s neuropathic pain was remarkably reduced and he avoided the indicated extremity amputation. According to the authors, their technique can be improved, in order to achieve more satisfactory results in neurological recovery in such cases in the future (15). This study highlights the possible benefits of PRP in neurological recovery, but has to be enhanced with more meticulous research for more reliable conclusions.

Furthermore, Hibner et al. (16) described a surgical technique for the treatment of persistent pudendal neuralgia after failed initial surgical decompression. A transgluteal decompression of the pudendal nerve was performed in 10 patients with the above characteristics. After the adhesiolysis, the nerve was coated with PRP. This retrospective clinical trial (23 months mean follow-up) showed that eight out of nine patients reported global improvement, with two patients reporting complete resolution of symptoms. It was not obvious from this study the role of PRP – in comparison to the surgical adhesiolysis and painkillers that were injected through a pain buster catheter into the Alcock canal – in the percentage of clinical improvement. Moreover, the small number of the patients included in the study, as well as the fact that the trial was not prospective-randomized, emphasizes the need for higher level of evidence papers to demonstrate the possible usefulness of PRP in such cases (16).

Finally, Scala et al. performed a clinical randomized trial (17) in which 20 patients who underwent a superficial parotidectomy were separated in two groups: in the first, PRP gel was used at the surgical site, in contrast with the second. The authors concluded that the use of PRP in patients who underwent superficial parotidectomy may play – among other positive effects – a protective role against neurological deficit of facial nerve. However, because of the relatively small number of patients that participated in the study, trials of higher quality are needed to support these findings.

## Conclusion

The variety of platelet enriched plasma’s clinical use in every day’s practice seems to be, at least, impressive, but the evidence-based recommendations are often equivocal. We have been able to identify 29 clinical trials – Level I, II, or III-investigating the possible clinical benefits of PRP in different orthopedic related diseases or conditions. The potential clinical use of PRP in spinal fusion, bone formation, chondropathy, osteoarthritis, lateral epicondylatis among other tendinopathies, muscle, ligamentous or tendinous injuries, and skin wounds has been investigated. Surgical applications of PRP in ACL reconstruction, rotator cuff repair, Achilles tendon tear, or non-unions show promising results in some papers and discouraging in others. The researcher’s field appears to be wide for PRP, but the image remains blur.

For some authors – Level I studies – PRP is suggested as a new effective treatment in cartilage diseases, like knee osteoarthritis (18, 19). Moreover, there are papers proposing PRP as a first-line therapy in osteochondral lesions (20). PRP guided injections have proven to be efficient in chronic lateral epicondylitis (21–23). The results in other tendinopathies, such as jumper’s knee (24) or Achilles tendinopathy (25), are not as clear (26). In a literature review of eight controlled clinical trials was concluded that the use of platelet concentrates into the femoral channel of ACL reconstruction (hamstring’s tendons) probably leads to a 20–30% higher rate of graft maturation (27). Overall, there is a tremendously growing demand for clinical studies involving PRP effects in several tissues, from bone to ligaments and tendons. But what evidence do we have about the area of peripheral nerves?

According to the International cellular medicine society (PRP guidelines) (28), entrapment neuropathies that have failed conservative treatment may be treated by percutaneous release of nerves using PRP. This society concludes this section with the encouragement of further investigation on this area.

We have been able to identify two randomized trials regarding the clinical application of PRP in peripheral neuropathies. Two other studies had a level of evidence 5, and one had a level of evidence 4. Besides, there are several techniques of preparation of PRP (29) and different anatomic areas of application, while the optimal dosage, frequency of administration, and appropriate stage of neuropathy for the use of PRP have not been investigated.

Although there are many published (*in vivo* or *in vitro*) animal studies (and some anecdotal clinical reports) about the use of PRP in peripheral neuropathies, literature cannot yet give a convincing answer about the value of this therapeutic strategy. Nevertheless, results of the so-far published clinical studies are encouraging. Consequently, it is obvious that this interesting field of research gives to the scientists the ability to expand it extensively, in terms of both quality and quantity. It is obvious that more clinical trials are needed to yield more secure evidence regarding the beneficial effect of PRP usage in peripheral neuropathies.

## Conflict of Interest Statement

The authors declare that the research was conducted in the absence of any commercial or financial relationships that could be construed as a potential conflict of interest.
